# Sexual Dimorphism in Teeth Dimension and Arch Perimeter of Individuals of Four Ethnic Groups of Northeastern India

**DOI:** 10.7759/cureus.37905

**Published:** 2023-04-20

**Authors:** Plabita Mazumder, Hema Bahety, Alaka Das, Putul Mahanta, Dipanjal Saikia, Ranjumoni Konwar

**Affiliations:** 1 Dentistry, Lakhimpur Medical College and Hospital, Lakhimpur, IND; 2 Community Medicine, Lakhimpur Medical College, Lakhimpur, IND; 3 Biochemistry, Assam Medical College, Dibrugarh, IND; 4 Forensic Medicine and Toxicology, Nalbari Medical College, Nalbari, IND; 5 Dentistry, Assam Medical College and Hospital, Dibrugarh, IND; 6 Radiology, State Cancer Institute (SCI) Gauhati Medical College and Hospital, Gauhati, IND

**Keywords:** odontometry, permanent teeth, arch perimeter, mesiodistal dimension, sexual dimorphism

## Abstract

Objectives

There is a disparity between males and females when it comes to size and appearance. In forensic and anthropological investigations, it is crucial to identify an unknown individual's gender, and it is possible to discern individual differences based on differences in dental features among populations. For identifying individuals, tooth dimensions are an effective, simple, and low-cost means of determining sex. Based on dental casts, this study aims to assess sexual dimorphism among four ethnic tribes of Northeast India using the mesiodistal (MD) dimension of canines and arch perimeter (AP) of the upper and lower jaws.

Methods

In each of the four ethnic groups studied, 50 males and 50 females with dental casts were measured in millimetres for the MD dimension of canines and AP of the upper and lower jaws. SPSS version 20 (IBM Corp., Armonk, NY) was used to analyze the data based on Student's t-test, considering a p-value of <0.05 as significant.

Results

Males had significantly larger MD dimensions of canines in the maxillary and mandibular jaws (p-value<0.05). The AP of both maxilla and mandible are higher in males than females in all four ethnic groups. However, the difference between the two genders for the AP of the maxilla is statistically significant only in Meitei and Singpho groups (p-value<0.05). In the case of the mandibular jaw, the AP was significantly lower in females in all four ethnic groups (p-value<0.05).

Conclusion

Significant sexual dimorphism exists among the individuals in the four ethnic group populations. The MD dimension and AP are essential to establish sexual dimorphism among populations. The MD dimension of the maxillary and mandibular canine and AP showed significant sexual dimorphism in the present study among all four ethnic groups.

## Introduction

The field of dental anthropology was established to acknowledge the expanding volume of literature describing the most indestructible and morphologically revealing portion of the human skeleton, the teeth. Though it is hard to attribute any specific anatomical feature to a specific race, a detailed analysis of physical, skeletal, and dental characteristics may collectively support an individual's racial identity [1].

Identifying an individual is a pre-requisite for death certification and personal, social, and legal reasons. Comparing tooth measurements in males and females or comparing non-metric dental attributes is used to determine gender using dental features. In times of mass disasters, where the remains are typically destroyed beyond recognition, morphometrics plays a crucial role in ascertaining the gender [2] of an individual.

Determining sex by tooth dimension is quick, simple, and inexpensive, and it can also help to identify individuals. However, because sexual dimorphism varies by population, sex differentiation from odontometric data necessitates population-specific norms [3]. Various research conducted worldwide demonstrates the usefulness of odontometric in gender determination [2-17].

The states of Northeast India are home to a diverse range of tribes and ethnicities with a pure genetic pool, with 60%-70% of the population being tribal and Mongoloid. No odontometric study has yet been conducted in this region. Hence, the current study is undertaken among these four ethnic groups in this region to assess the efficacy of odontometric data in determining the gender of an individual using the mesiodistal (MD) dimensions of canine and arch perimeter (AP) using dental casts.

## Materials and methods

The study was conducted at the Oral Pathology and Microbiology department, Kothiwal Dental College and Research Centre, Moradabad. The study comprised people aged 20 to 30 years with a complete set of healthy teeth and gingiva up to the second molar. Persons with significant loss of tooth substance owing to attrition, caries, or restoration were discarded from the study. Also, the study did not include persons who have undergone orthodontic treatment. An oral examination of the participants was done before including them in the study. Informed consent was obtained from the study participants. The ethics committee of Kothiwal Dental College and Research Centre approved the current research vide Ref No KDCRC/IERB/OP/2014/23.

Study population and sample

The four ethnic groups included in the present study are Miching, Meitai, Ao, and Singpho. Miching is from Assam's second most populous scheduled tribe. They are all blood brothers with various social constraints in their marital connections. Nagaland's Ao is a dominant ethnic group with its dialect. Meitei is Manipur's most populous ethnic group, accounting for more than 60% of the state's population. Singpho is a member of the Burmese Kachin tribe inhabiting Arunachal Pradesh's Lohit and Changlang districts.

Using a simple random sampling method, 50 male and 50 female subjects aged 20-30 years from each of the four ethnic groups under study were selected. Each participant's dental castings were measured using a digital vernier caliper to obtain both jaws' MD dimensions of the canines and AP. The data obtained were recorded in a separately prepared custom-made record sheet.

Tooth dimension measurement

Alginate imprints for the upper and lower arches were taken in appropriate perforated trays for each participant. Impressions were promptly cast with high-quality dental stones to avoid problems with model distortions. The MD diameter of the crown was determined by measuring the most significant distance between the approximated surface of the crown with a sliding caliper with pointed tips parallel to the occlusal and vestibular surfaces of the crown. AP was measured with a soft brass wire. The wire was first contoured to the particular arch form, which, in the posterior region, starts from the mesial to the first molar and then crosses the contact points. At the same time, anteriorly, it follows the incisal edges and ends again on the mesial surface of first molar of the opposite side. However, in the case of proclined incisors, the wire would touch the cingulum of the incisor teeth, and if the incisors were reclined, then it would touch the labial surfaces of the incisors.

Statistical analysis

The data were analysed using the SPSS version 20 (IBM Corp., Armonk, NY). Quantitative variables were presented as mean and standard deviation (SD). Differences between mean values were tested using Student's t-test or their non-parametric complements depending upon the normality of the data. The cut-off p-value was considered 0.05 for a statistically significant test.

## Results

The highest mean MD dimension in maxillary canine was observed among males of the Singpho group (7.65±0.19), while the least was observed among the Miching group in both genders with 7.16±0.20 in males and 6.58±0.41 in females. The MD dimension of the maxillary canine of males is higher than females and was found to be significantly different between the two genders in all four ethnic groups (p-value<0.01). In the case of mandibular canines, the highest MD dimension was found among the Meitei group (6.78±0.53) while the least was among females of the Ao group (5.96±0.47). In the mandibular canines, males were found to have significantly higher MD dimensions than females in all four groups (Table 1).

**Table 1:** Mesiodistal (MD) dimension of canines in the four ethnic groups SD: standard deviation

Teeth	Observations	Ethnic Groups							
		Ao		Meitei		Miching		Singpho	
		Male	Female	Male	Female	Male	Female	Male	Female
Maxillary Canine	Mean	7.33	6.60	7.45	6.84	7.16	6.58	7.65	6.70
	SD	0.34	0.38	0.21	0.27	0.20	0.41	0.19	1.14
	p-value	<0.0001		<0.0001		<0.0001		0.0016	
Mandibular Canine	Mean	6.38	5.96	6.78	6.00	6.41	6.04	6.68	6.04
	SD	0.38	0.47	0.53	0.31	0.59	0.40	0.34	0.28
	p-value	0.0039		<0.0001		0.0241		<0.0001	

The highest mean AP in the maxilla and mandible was observed among the Meitei group in both genders, as shown in Table 2.

**Table 2:** Arch perimeter (AP) of teeth among the four ethnic groups SD: standard deviation

Arch perimeter (AP)	Observations	Ethnic Groups							
		Ao		Meitei		Miching		Singpho	
		Male	Female	Male	Female	Male	Female	Male	Female
Maxilla	Mean	7.80	7.60	7.99	7.64	7.78	7.52	7.68	7.38
	SD	0.19	0.42	0.32	0.37	1.14	0.41	0.26	0.17
	p-value	0.064		0.003		0.348		0.000	
Mandible	Mean	6.85	6.35	6.86	6.42	6.86	6.35	6.78	6.25
	SD	0.40	0.34	0.40	0.22	0.35	0.38	0.47	0.17
	p-value	0.0001		0.0002		0.0001		0.0001	

The Singpho females exhibited the least AP in the maxillary (7.38±0.17) and mandibular jaw (6.25±0.17). AP of both maxilla and mandible are higher in males than females in all four ethnic groups. However, the difference between the two genders for AP of the maxilla is statistically significant only in Meitei and Singpho groups. The difference between the two genders for the AP of the mandible is statistically significant (p-value<0.05) in all the ethnic groups.

## Discussion

The present study observed that sexual dimorphism in the maxillary and mandibular canines was highly significant in all four ethnic groups. The present study's result agrees with other studies [3-10,12]. Various studies suggested that the maxillary canines are the most significant tooth in gender determination [2,16], similar to our study except for the Singpho group. Another study conducted among ethnic groups of Southern India reported the MD dimensions of the mandibular canine as the most sexually dimorphic measurement [17] except for the Ao, Miching group.

Canines are distinct from other teeth in terms of survival and sex division. These distinctions are most likely due to their function, which differs from those of other teeth in evolution. As per different postulates of evolutionary scientists, the shift of aggressive function from canines in apes to fingers in humans occurred during primate evolution. Until this transfer was complete, survival depended on the canines, particularly those of males. Mandibular canines are the teeth with the most sex differences in size, prominence, and eruption age in modern humans [18]. Also, as the influence of the Y chromosome is not uniform in all teeth, it makes canines notably significant in determining sex [19]. 

In the present study, considering the AP of the maxilla, it was observed that the values in males were higher than in females in all four ethnic groups. However, the difference between the male and female dimensions is statistically significant only in Meitei and Ao groups. Regarding the AP of the mandible, it was observed that the values in males were higher than in females in all four ethnic groups, and the difference between males and females is statistically significant in all four ethnic groups. The results of our study significantly establish sexual dimorphism in AP in the Northeast population. These results agree with similar other studies [20-23]. A recent study established a positive correlation between AP and MD dimensions of canines [24]. However, the result of the present study differs from other studies [25-27].

Males usually have a longer time of amelogenesis than females, which results in thicker enamel. Furthermore, in deciduous and permanent dentition, females complete crown calcification earlier than boys. Also, the genetic linkage of X and Y chromosomes and hormonal influences directly affect tooth size in males and females [28]. The dentine region is larger in male teeth than in female teeth, which also influences the diameter of the tooth crown [29]. Also, biological variation, a characteristic of life attributed to family, genetics, and environmental factors, affects tooth size.

During the early bell stage, before the calcification of teeth begins, the cusp of the tooth development process begins to take shape and go through a process controlled by activators and inhibitors, creating enamel knots. The gene that codes for activators and inhibitors and governs their expression modulates the rate and amount of enamel deposition. This genetic influence causes some but not all teeth to be dimorphic between genders [30].

Although the ethnic and tribal population of Northeast India shares a common genetic pool and racial identity of Mongoloid origin, the findings of the present study indicate that there are differences in odontometric features in each of the studied ethnic group populations, even within the same population. Therefore, it is necessary to determine population-specific values to make identification possible based on dental measurements among these populations.

Limitation

The present study included only four ethnic groups of Northeast India. Also, only the MD dimension of the canines and the AP of the jaws were considered for comparing gender dimorphism. However, a broader study including more tribal populations and other dental dimensions like canine index measurements may provide a more elaborated insight into identifying the variability and sexual dimorphism among those populations.

## Conclusions

Comparing the MD dimension of canines and AP of jaws between males and females of the four ethnic groups suggests significant sexual dimorphism. The data on teeth dimensions thus may be helpful in sex determination among the ethnic groups studied. Also, the study emphasises the necessity of population-specific odontometric data of the ethnic groups of Northeast India for forensic odontology, dental anthropology, and routine dental practice for providing high-quality population-specific dental care and treatment planning.
